# VA Compassionate Contact Corps: A Phone-Based Intervention for Veterans Interested In Speaking With Peers

**DOI:** 10.1093/geroni/igab046.788

**Published:** 2021-12-17

**Authors:** Jennifer Sullivan, Lisa Gualtieri, Maura Campbell, Heather Davila, Jacquelyn Pendergast, Prince Taylor

**Affiliations:** 1 VA Providence Medical Center LTSS COIN and Brown University, Providence, Rhode Island, United States; 2 VA, Syracuse, New York, United States; 3 Department of Veterans Affairs, St. Lois, Missouri, United States; 4 Iowa City VA Healthcare System; University of Iowa Carver College of Medicine, Iowa City, Iowa, United States; 5 CHOIR, VA Boston healthcare System, Boston, Massachusetts, United States; 6 Department of Veterans Affairs, Washington, District of Columbia, United States

## Abstract

The VA Voluntary Service has developed and implemented a new social prescription program called Compassionate Contact Corps which was created during the COVID-19 pandemic when in-home volunteers could no longer enter Veterans’ homes. The program targets Veterans who are lonely, socially isolated or seeking additional social connection. Volunteers and Veterans are matched based on common interests. Trained volunteers provide support by making periodic phone calls. Program referrals are made from VA providers in several clinical programs (e.g. Home-based Primary Care). To date, CCC has been implemented in more than 80 sites in the VA, with 310 volunteers, 5,320 visits, and 4,757 hours spend with Veterans.

